# Guinea Worm Disease Presenting as a Subcutaneous Calcification

**DOI:** 10.7759/cureus.62733

**Published:** 2024-06-19

**Authors:** Virendra Athavale, Guneet Singh, Siddharth Tiwari, Kondapalli Sri Sai Teja Sampath

**Affiliations:** 1 General Surgery, Dr DY Patil Medical College and Hospital, Pune, IND; 2 General Surgery, Dr DY Patil Vidyapeeth, Pune, IND

**Keywords:** calcified subcutaneous mass, dracunculiasis, keloid, calcifications, guinea worm

## Abstract

A female patient in her early 50s presented with a complaint of multiple right thigh swellings for eight years which on evaluation were found to be guinea worms. The patient had no risk factors as she was living in a rural area of Maharashtra. We performed radiological investigations which only revealed calcifications in subcutaneous planes. On exploring the excised specimen post-surgery there was a worm-like appearance which on histopathological study confirmed guinea worm disease.

We highlight this unusual presentation of guinea worm disease so that surgeons can treat the condition effectively.

## Introduction

Guinea worm disease (GWD), commonly known as Dracunculiasis, is a neglected tropical disease caused by the parasite *Dracunculus medinensis.*

GWD infestations have been known to cause infections in humans since antiquity. The Bible refers to it as a "fiery serpent" [1]. It is a parasitic disease spread through contaminated water primarily affecting the rural areas of South Asian and African underdeveloped countries without access to clean drinking water. The latest incidence in India was recorded in July 1996, although three further cases from various sections of Rajasthan were later reported [2].

Once dead, the adult worm may develop calcification, which typically occurs in the lower extremities and usually has the appearance of a long, serpiginous string [3].

## Case presentation

A female patient in her early 50s from rural Maharashtra state, India, presented to the general surgery department of a tertiary care centre with the complaint of multiple swellings in the right thigh for eight years. She had no history of trauma, pain, discharge, itching, or restriction of movements, and no evidence of similar swellings in the body. The patient was non-diabetic and non-hypertensive.

On examination, there was evidence of multiple swellings, with the largest measuring 3x3cm with a smooth surface. Its borders appeared to be regular and there were no scars or sinuses.

**Figure 1:**
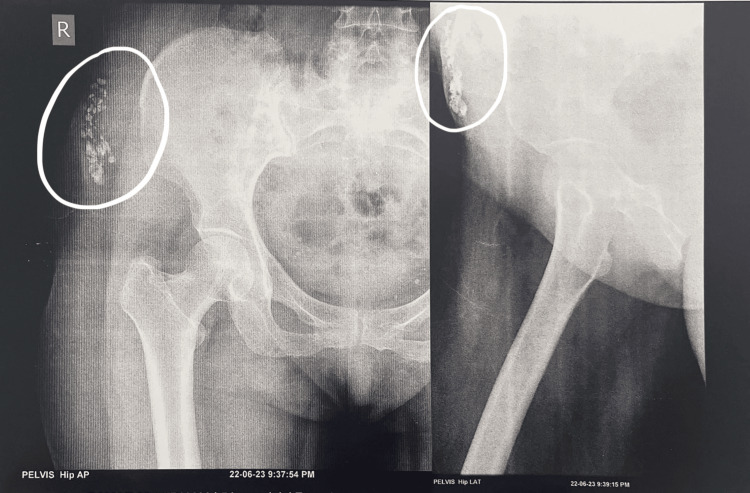
Subcutaneous, radio-opaque, beaded rice-grain lesions

On palpation, the swellings were hard, gritty in consistency, non-tender, on the subcutaneous plane, and movements were restricted (Figure 1).

**Figure 2:**
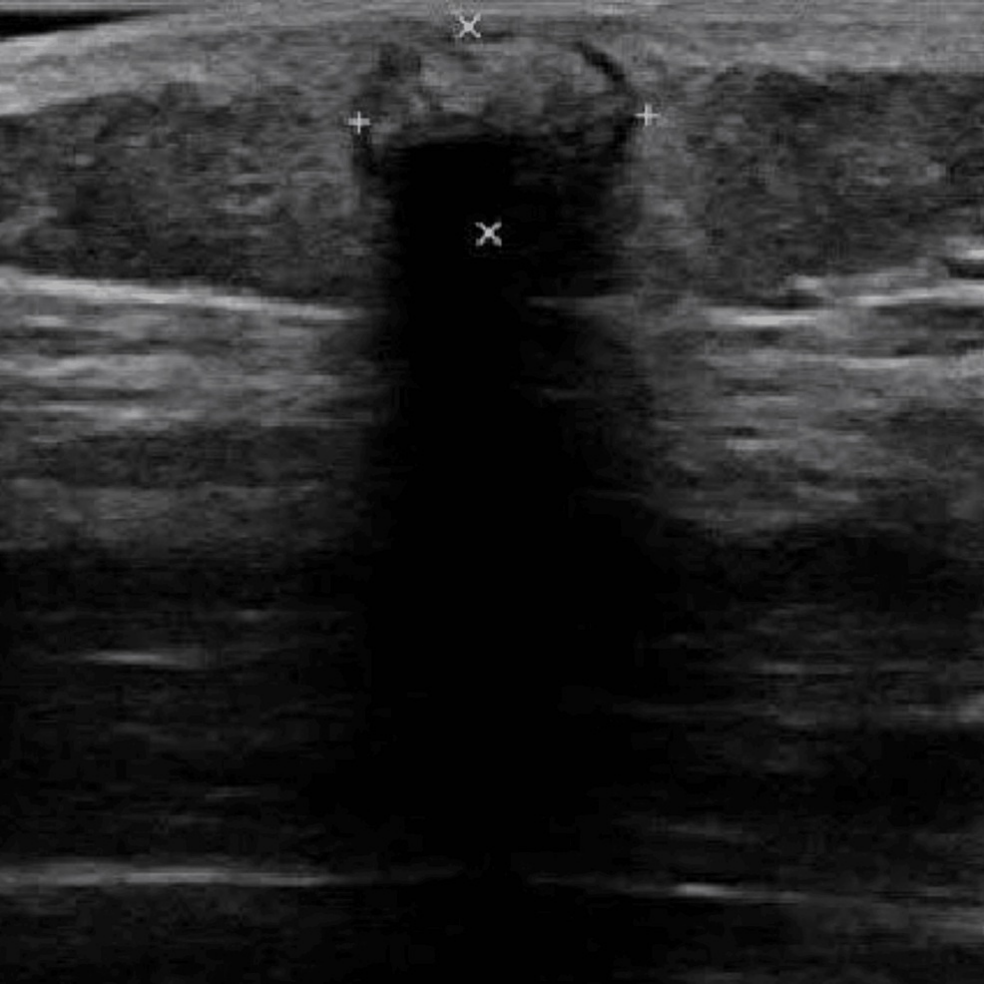
Ultrasound image showing hypoechoic region with posterior acoustic shadowing, indicative of foreign body or calcified structure within the tissue

On radiological examination, radio-opaque beaded lesions resembling rice grains were seen in the subcutaneous plane of the right thigh (Figure 2).

**Figure 3:**
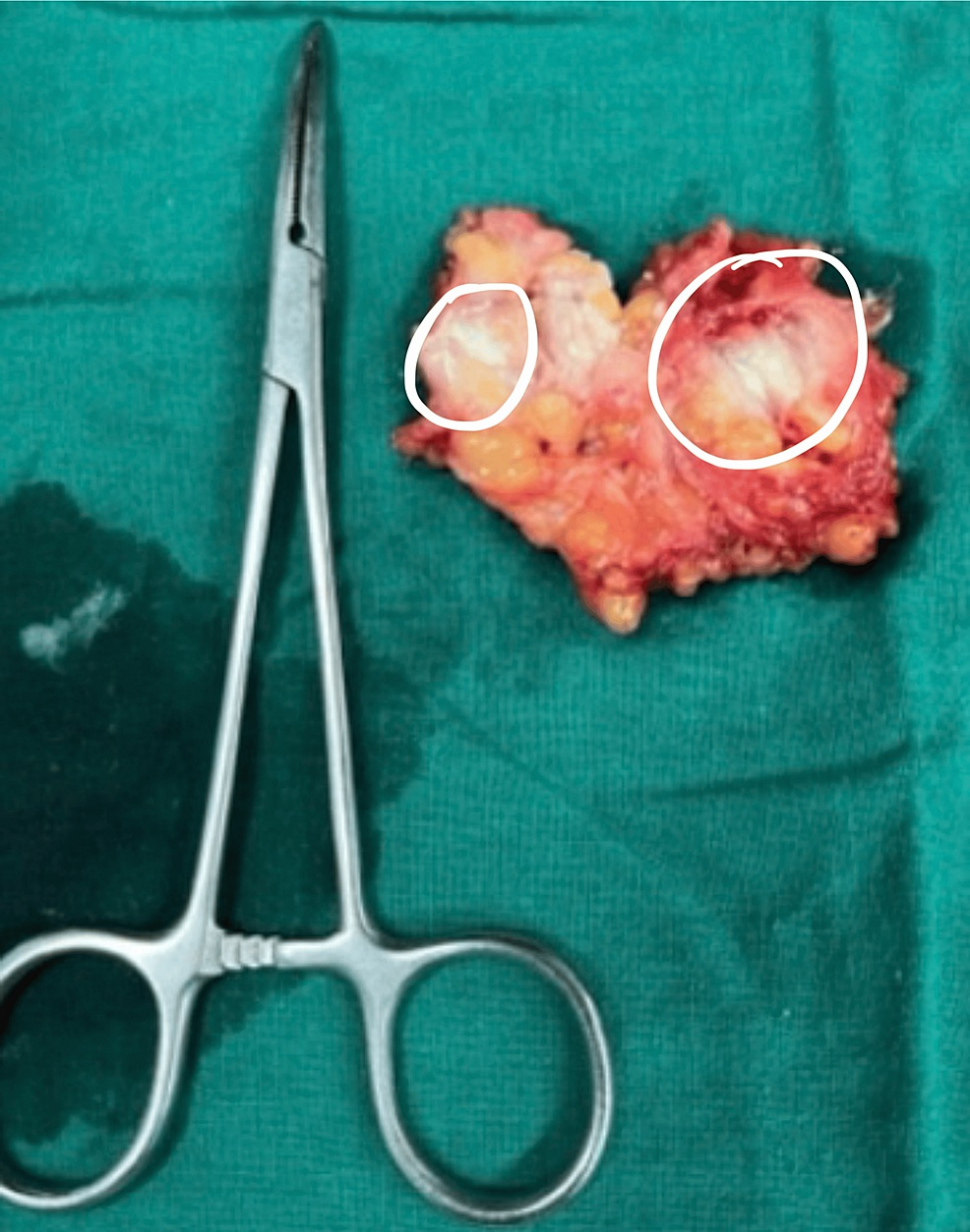
Circled areas in excised specimen showing calcified guinea worms

On ultrasonography (USG), the subcutaneous foreign body of calcifications was noted in the subcutaneous plane in the right hip region. Soft tissue appeared normal, there was no evidence of fluid in tissue or tendons or soft tissue oedema. After obtaining the consent of the patient, excision and excision of multiple subcutaneous calcifications was planned under spinal anaesthesia and the excised specimen was sent for histopathology examination for definitive diagnosis (Figure 3).

**Figure 4:**
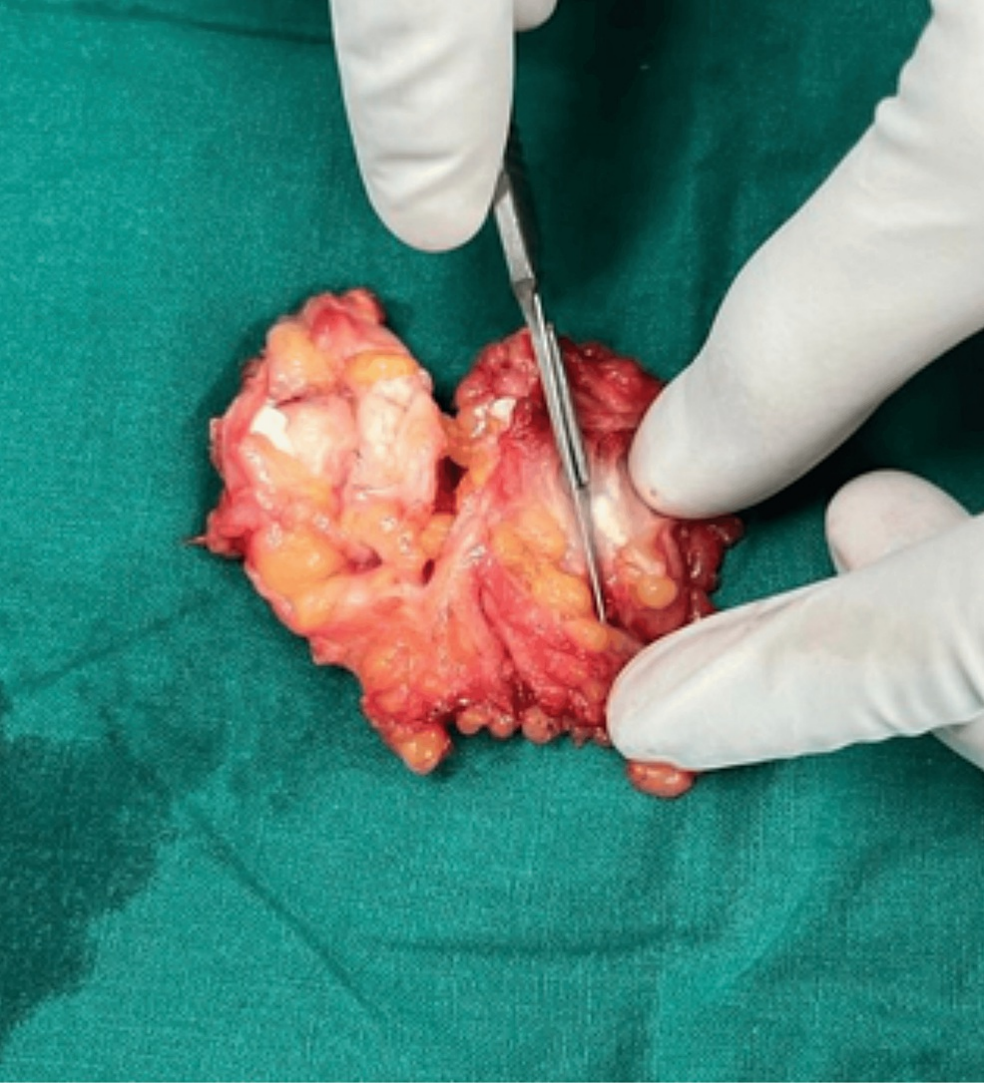
Excised specimen in total with naked eye appearance of calcified Guinea worms

A naked-eye examination yielded evidence of calcified worms on the excised specimen (Figure 4).

**Figure 5:**
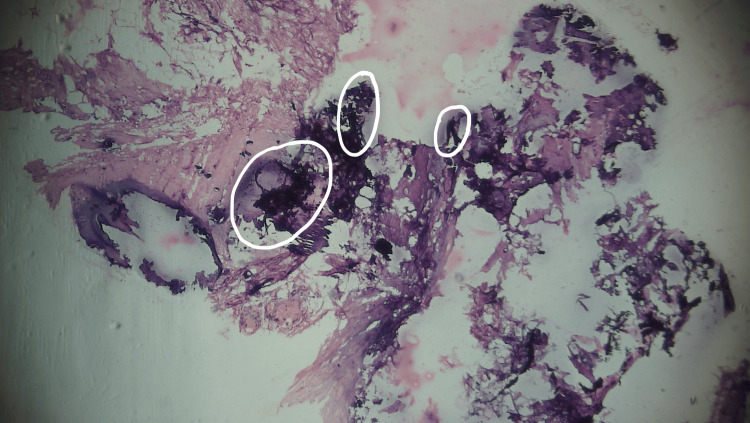
Circled areas in the histopathology specimen showing dead necrotic tissue with eosinophilic calcified lesions

**Figure 6:**
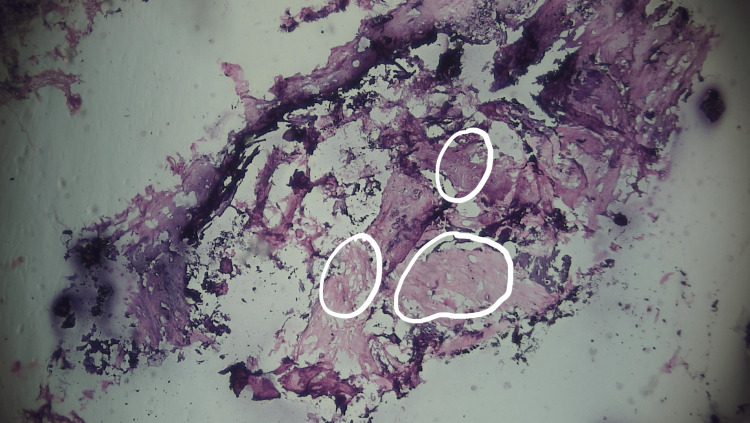
Circled areas showing fibrofatty tissue in excised specimen

**Figure 7:**
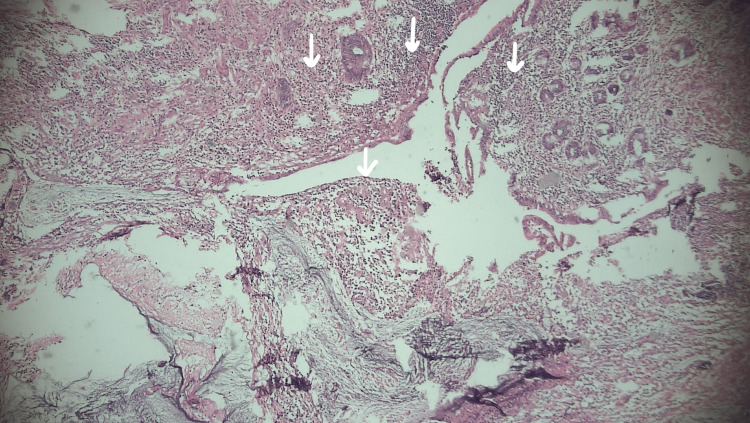
Arrows indicating lymphocytic infiltration in overlying fibrofatty tissue

Surgical excision of the subcutaneous calcifications was done under spinal anaesthesia and the excised specimen was sent for histopathological examination for definitive diagnosis (Figures 5-7). 

On histopathological gross examination, the skin was covered with fibrofatty tissue. Lymphocytic infiltration and multiple instances of granular, eosinophilic calcified material of various sizes were noted.

## Discussion

Dracunculiasis is considered eradicated from India. Guinea worm disease (GWD), also known as dracunculiasis, is a parasitic condition brought on by the parasitic worm *Dracunculus medinensis*, commonly known as the guinea worm. Consuming stagnant water with copepods harbouring guinea worm larvae is the mechanism of transmission. When someone drinks such stagnant water, they become infected with guinea worms. Copepods that reside in these stagnant water sources consume guinea worm larvae. Before they can infect people, the larvae in copepods take two weeks to reach maturity. After ingestion, these copepods perish and immediately expel their larvae. These expelled larvae later pierce the stomach and intestinal wall of the host and finally enter into the abdominal cavity and retroperitoneum. The male worms die after reaching adulthood and copulation, and the females migrate to the subcutaneous tissues towards the skin's surface. They vary in size from between 70 and 120 centimetres. Approximately a year after infection, the female worm triggers a skin blister, usually on the distal lower extremities, which erupts when it comes into contact with water, which the patient seeks out to ease the discomfort in the area. In the water, the female worm emerges and spews larvae. A copepod consumes the larvae to complete the life cycle [4].

The worm migrates into subcutaneous tissues after penetrating the gastrointestinal mucosa. While some worms (often gravid females) lodge in subcutaneous tissue, perish, and become encapsulated and calcified, others emerge through skin lesions. The mature female worm may result in calcification, acute aseptic arthritis, a skin blister, or a sterile abscess [5].

Even though radiological diagnoses are typically sporadic, it is still within the realms of possibility. In the soft tissues, radiography typically reveals a recognizable long, string-like, serpiginous calcified lesion. Nevertheless, morphological differential diagnosis should rule out other parasite diseases. Much smaller calcifications found on the hands indicate Loa loa and Onchocerca volvulus, whereas numerous "rice grain" calcifications found along muscle fibres point to cysticercosis. These radiological characteristics typically lead to an aetiological diagnosis. When the worm has partially decomposed and the calcifications are amorphous, diagnostic issues can arise [6-8]. Ectopic locations for calcified worms include the pericardium, orbit, and central nervous system [9].

A mild temperature, an itchy rash, nausea, vomiting, diarrhoea, and lightheadedness are among the clinical symptoms. Most frequently, a blister appears on a lower extremity. It grows and produces excruciating agony and a burning feeling, which is relieved when the affected area is immersed in water. Cellulitis, abscesses, septic shock, and septic arthritis are examples of problems that might appear in the acute stage. Joint abnormalities and calcification of the worm can appear in the late stage. Treatment for the acute stage includes local ulcer care as well as gradual worm removal. To stop secondary infection, antibiotics are administered systemically. Rarely, the calcified worm may produce repeated issues in the chronic stage, as in our case, and require surgical removal.

## Conclusions

Despite being long thought to be eradicated in India, calcified guinea worms might occur occasionally in patients.

In order to make an early diagnosis and begin treatment, doctors should be aware of this uncommon condition and should have a high index of suspicion for the same. If the calcified worm repeatedly causes issues, it should be surgically removed.
